# Therapeutics targeting angiogenesis: Genetics and epigenetics, extracellular miRNAs and signaling networks (Review)

**DOI:** 10.3892/ijmm.2013.1444

**Published:** 2013-07-16

**Authors:** MASARU KATOH

**Affiliations:** Division of Integrative Omics and Bioinformatics, National Cancer Center, Tokyo 104-0045, Japan

**Keywords:** bevacizumab, sunitinib, sorafenib, dovitinib, ponatinib, AZD4547, breast cancer, lung cancer, colorectal cancer, gastric cancer, field cancerization, poly(lactic-co-glycolic acid), chitosan, vascular medicine

## Abstract

Angiogenesis is a process of neovascular formation from pre-existing blood vessels, which consists of sequential steps for vascular destabilization, angiogenic sprouting, lumen formation and vascular stabilization. Vascular endothelial growth factor (VEGF), fibroblast growth factor (FGF), angiopoietin, Notch, transforming growth factor-β (TGF-β), Hedgehog and WNT signaling cascades orchestrate angiogenesis through the direct or indirect regulation of quiescence, migration and the proliferation of endothelial cells. Small-molecule compounds and human/humanized monoclonal antibodies interrupting VEGF signaling have been developed as anti-angiogenic therapeutics for cancer and neovascular age-related macular degeneration (AMD). Gene or protein therapy delivering VEGF, FGF2 or FGF4, as well as cell therapy using endothelial progenitor cells (EPCs), mesenchymal stem cells (MSCs) or induced pluripotent stem cells (iPSCs) have been developed as pro-angiogenic therapeutics for ischemic heart disease and peripheral vascular disease. Anti-angiogenic therapy for cancer and neovascular AMD is more successful than pro-angiogenic therapy for cardiovascular diseases, as VEGF-signal interruption is technically feasible compared with vascular re-construction. Common and rare genetic variants are detected using array-based technology and personal genome sequencing, respectively. Drug and dosage should be determined based on personal genotypes of *VEGF* and other genes involved in angiogenesis. As epigenetic alterations give rise to human diseases, polymer-based hydrogel film may be utilized for the delivery of drugs targeting epigenetic processes and angiogenesis as treatment modalities for cardiovascular diseases. Circulating microRNAs (miRNAs) in exosomes and microvesicles are applied as functional biomarkers for diagnostics and prognostics, while synthetic miRNAs in polymer-based nanoparticles are applicable for therapeutics. A more profound understanding of the spatio-temporal interactions of regulatory signaling cascades and advances in personal genotyping and miRNA profiling are required for the optimization of targeted therapy.

## 1. Introduction

Angiogenesis is a process of neovascular formation from pre-existing blood vessels during embryogenesis, adult tissue homeostasis and carcinogenesis (1–4). As blood vessels are involved in the delivery of oxygen and nutrients, as well as in the withdrawal of carbon dioxide and metabolic waste from the whole body, the dysregulation of angiogenesis causes a variety of diseases. Vascular malformations, DiGeorge syndrome and hereditary hemorrhagic telangiectasia, are congenital disorders associated with abnormal angiogenesis. Cancer, neovascular age-related macular degeneration (AMD) and diabetic retinopathy are caused or promoted by aberrant angiogenesis, whereas ischemic diseases of the heart, brain and limbs are caused by insufficient angiogenesis or vessel obstruction (1).

Therapeutic modalities targeting angiogenesis are divided into anti-angiogenic therapy for cancer and neovascular AMD, as well as pro-angiogenic therapy for cardiovascular diseases. In this review, the physiology of angiogenesis is briefly summarized to obtain a bird’s-eye view of this field. Subsequently, anti- and pro-angiogenic therapy is also reviewed to obtain a ‘frontal’ and ‘rear’ view of angiogenesis, respectively. Based on the three dimensional view of angiogenesis research, future directions of vascular medicine are also discussed.

## 2. Physiology of angiogenesis

Vascular endothelial cells, constituting the luminal surface of the inner vessel wall, are leading players in angiogenesis, while pericytes and the basement membrane, associating with and coating endothelial cells, are supporting players of angiogenesis (5). Angiogenesis occurs in multiple steps as follows: i) vascular destabilization induced by degradation of the basement membrane and decreased adhesion of endothelial cells; ii) angiogenic sprouting resulting from the migration of endothelial tip cells and the proliferation of endothelial stalk cells; iii) lumen formation by endothelial cells and the recruitment of pericytes to the surrounding region of the endothelial lumen; iv) vascular stabilization depending on tight junctions and basement membrane (1).

Vascular endothelial growth factor (VEGF), fibroblast growth factor (FGF2), angiopoietins (ANGPT1 and ANGPT2), Notch ligands [jagged 1 (JAG1) and Delta like ligand 4 (DLL4)] and transforming growth factor-β (TGF-β) regulate angiogenesis through their receptors on vascular endothelial cells. VEGF activates the eNOS, SRC, RAS-ERK and PI3K-AKT signaling cascades through VEGFR2 receptor on endothelial cells, which induce vascular permeability, endothelial migration, proliferation and survival, respectively (6,7). FGF2 promotes angiogenesis directly through FGFR1 receptor on endothelial cells via signaling cascades similar to VEGF, or indirectly through VEGF secretion from endothelial cells, cardiomyocytes and stromal cells (8). ANGPT1, secreted from pericytes, activates TEK/TIE2 receptor to maintain endothelial quiescence or stabilization, whereas ANGPT2, secreted from endothelial cells themselves by VEGF or hypoxia signaling, inhibits TEK to promote endothelial activation or sprouting (9). JAG1-Notch signaling promotes angiogenic sprouting, whereas DLL4-Notch signaling inhibits angiogenic sprouting (10). TGF-β signaling through TGFBR1/ALK5 receptor to the Smad2/3 cascade inhibits endothelial cell activation, maintaining endothelial quiescence, whereas TGF-β signaling through the ACVRL1/ALK1 receptor to the Smad1/5 cascade promotes the migration and proliferation of endothelial cells (5). The VEGF, FGF, Notch and TGF-β signaling cascades are directly involved in the angiogenic signaling of endothelial cells.

The VEGF, FGF, Notch and TGF-β signaling cascades cross-talk with WNT and Hedgehog signaling cascades to constitute the stem-cell signaling network (11,12). Indeed, DVL2-binding deubiquitinase FAM105B regulates WNT signaling and angiogenesis (13), while Hedgehog signaling is involved in the regulation of liver sinusoidal endothelial cells (14). FGF, Notch and canonical WNT signaling are involved in cell-fate determination based on mutual transcriptional regulation, whereas FGF, Notch, TGF-β, Hedgehog and non-canonical WNT signaling are involved in epithelial-to-mesenchymal transition (EMT) due to the upregulation of SNAI1 (Snail), SNAI2 (Slug), ZEB1, ZEB2 and TWIST (15). EMT is a cellular process similar to endothelial-to-mesenchymal transition (EndMT). Hypoxia induces angiogenesis as a result of VEGF upregulation (16) and controls cancer stem cells and EMT transition through the stem-cell signaling network (17). Angiogenesis is orchestrated by the VEGF, FGF, Notch, TGF-β, Hedgehog and WNT signaling cascades, which directly or indirectly regulate the quiescence, migration and proliferation of endothelial cells.

## 3. Anti-angiogenic therapy

Human/humanized monoclonal antibodies and small-molecule inhibitors targeting VEGF and VEGFRs have been developed as anti-angiogenic therapeutics. Bevacizumab (Avastin) is a humanized anti-VEGF monoclonal antibody, which is applied for the treatment of patients with metastatic colorectal cancer (18), non-small cell lung cancer (NSCLC) (19), ovarian cancer and other types of cancer (20,21). Sunitinib (Sutent) (22) and sorafenib (Nexavar) (23) are multi-kinase inhibitors, targeting VEGFR2 and other protein kinases. Sunitinib is applied for the treatment of renal cell carcinoma (RCC) and gastrointestinal stromal tumor (GIST), while sorafenib is applied for the treatment of RCC and inoperable hepatocellular carcinoma (HCC) (24).

The most important clinical problem associated with cancer therapy using VEGF- or VEFGR-targeting agents is drug resistance, as a result of clonal expansion or subclonal evolution of tumors with the upregulation of other angiogenic factors, such as FGF2 (15,25). Dovitinib (TKI258) (26), ponatinib (AP24534) (27) and AZD4547 (28) are multi-kinase inhibitors, targeting VEGFR2 and FGFRs. FGFRs are aberrantly activated in cancer cells due to gene amplification, translocation, point mutation or other regulatory signaling mechanisms. Since the dual inhibition of VEGFR and FGFR induces antitumor effects directly through cancer cells and indirectly through endothelial cells, VEGFR/FGFR dual inhibitors have been investigated in clinical trials for various types of human cancer (15).

Adverse effects associated with the systemic administration of VEGF- or VEGFR-targeting drugs, include hypertension, proteinuria, cardiac ischemia, cerebral thrombosis, hemorrhage and gastrointestinal perforation (29). Ranibizumab (Lucentis) is a derivative of bevacizumab with better penetration to the retinal pigment epithelium. To reduce the adverse effects of the VEGF- or VEGFR-targeting systemic therapy to non-cancerous patients, an intravitreal injection of bevacizumab or ranibizumab is applied for the treatment of neovascular AMD (30).

## 4. Pro-angiogenic therapy

Gene (31–34), protein (35,36) and cell therapies (37–39) have been developed as pro-angiogenic therapeutics to artificially reconstruct vasculature for patients with ischemic heart and peripheral vascular diseases.

Viral vectors derived from adenovirus, adeno-associated virus (AAV), herpes simplex virus (HSV) and lentivirus, as well as non-viral plasmid vectors are applicable for gene therapy ectopically, expressing pro-angiogenic factors such as VEGF and FGFs. Adenoviral vector expressing VEGF (AdVEGF121) (31), adenoviral vector expressing FGF4 (Ad5.FGF4) (32), plasmid vector expressing VEGF (phVEGF-A_165_) (33) and plasmid vector expressing FGF1 (NV1FGF) (34) have been investigated in clinical trials for the treatment of ischemic diseases; however, they have failed due to adverse effects or non-effectiveness.

The intravenous infusion or local arterial injection of recombinant VEGF, FGF1 or FGF2 have failed to show beneficial effects for the treatment of patients with ischemic diseases, as a result of a short half-life or instability (35). Polymer-based hydrogel technology was thus applied for the treatment of ischemic diseases, as polymers or copolymers, such as chitosan, alginate, gelatin and lactic-*co*-glycolic acid, can be cross-linked to form microparticles, nanoparticles or films for the delivery and controlled release of pro-angiogenic factors (35,36). Endothelial progenitor cells (EPCs), mesenchymal stem cells (MSCs) and adipose tissue-derived stem cells (ADSCs), as well as the VEGFR2-positive population of embryonic stem cells (ESCs) and induced pluripotent stem cells (iPSCs) are also applicable for the treatment of ischemic diseases, as these stem/progenitor cells are able to differentiate into endothelial cells and to secrete pro-angiogenic factors (37–39). However, large-scale randomized clinical trials are mandatory to demonstrate the effectiveness of the polymer-based protein therapy or stem/progenitor cell therapy for patients with ischemic diseases.

## 5. Conclusion

The clinical application of anti-angiogenic therapeutics for cancer and neovascular AMD has been more successful than that of pro-angiogenic therapeutics for cardiovascular diseases, since the interruption of angiogenic signaling is technically feasible compared with the re-construction of vascular structure.

## 6. Perspectives

Human diseases, such as cancer, cardiovascular diseases, hypertension and diabetes mellitus, occur and progress as a result of mutual interactions of genetic factors and environmental factors (40,41). Genetic factors include common variations and rare mutations caused by single nucleotide changes or germline copy number changes, while environmental factors include infectious agents, chemicals, X-rays, ultra-violet light and lifestyle habits, such as smoking, high calorie diet, high salt intake, low fruit/vegetable intake and infrequent exercise. Epigenetic changes and microRNA (miRNA) dysregulation have also been implicated in human diseases. Although the strategy and device used are significantly distinct in anti- and pro-angiogenic therapeutics, the effectiveness of both these therapeutic modalities is affected by genetic variations, epigenetic alterations and miRNAs. Genetics and epigenetics, miRNAs and signaling networks are discussed in the following section as issues to be further addressed.

The single nucleotide polymorphisms (SNPs), rs699947 (-2578C>A), rs833061 (-1498T>C) and rs2010963 (-634C>G), are located within the promoter region or 5′-untranslated region (UTR) of the *VEGF* gene. RCC patients with an ACG haplotype for the rs699947, rs833061 and rs2010963 SNPs have a risk of sunitinib-induced hypertension (42), whereas RCC patients with a CC genotype of rs699947, a TT genotype of rs833061 and a CC genotype of rs2010963 are the worst responders to sunitinib (43). The CC genotype of rs2010963 is associated with higher serum levels of VEGF protein in patients with proliferative diabetic retinopathy (44), and breast cancer patients with a higher plasma VEGF concentration are the worst responders to bevacizumab (45). The therapeutic and adverse effects of anti-angiogenic drugs targeting VEGF signaling cascades are affected by SNPs of the *VEGF* gene that regulate circulating VEGF levels. Common variants are genotyped by using array-based technology, while rare variants by using personal genome sequencing. Genotypes of the *VEGF* gene, as well as other genes encoding angiogenesis regulators should be utilized for the selection and dosage of anti- and pro-angiogenic drugs.

Epigenetics regulates lineage-specific or disease-associated transcription depending on DNA methylation, histone modifications or non-coding RNAs, rather than changes in the genomic DNA sequence itself (46,47). Chronic inflammation associated with epigenetic changes gives rise to various types of cancer, such as colorectal, breast and gastric cancer and is known as the concept of field cancerization (48–50). In addition, epigenetic alterations associated with smoking or environmental pollution are associated with the increased risk of cardiovascular diseases (47). These facts indicate that cancer and non-cancerous diseases occur from a field of epigenetic alterations. Polymer-based hydrogel film may be utilized for the delivery of drugs targeting epigenetic processes and angiogenesis for the pro-angiogenic therapy of cardiovascular diseases.

miRNAs are single-stranded RNAs of approximately 22 nucleotides in length, which are derived from non-coding primary miRNAs as a result of sequential processing by Drosha, Dicer and RISC. miRNAs bind to complementary sequences of target mRNAs through perfect or imperfect base-pairing, thus repressing target genes through direct cleavage or translational repression, respectively (12,51). miR-15, miR-16, miR-20a and miR-20b are anti-angiogenic miRNAs targeting *VEGF* mRNA, whereas the miR-17-92 cluster, miR-27b and let-7f are pro-angiogenic miRNAs (52,53 and references therein). A hot topic in this field are the circulating miRNAs in the blood plasma or serum, which are carried by nano-sized exosomes or micro-sized microvesicles (54,55). Expression profiles of exosomal miRNAs are applied for the differential diagnosis of schizophrenia and bipolar disorder (56), while microvesicles derived from EPCs are involved in kidney protection from ischemia-reperfusion injury in a miRNA-dependent manner (57). Circulating miRNAs in exosomes and microvesicles are functional biomarkers to be used for the diagnostics and prognostics of human diseases (58), while synthetic miRNAs in polymer-based hydrogel nanoparticles are applicable for therapeutics.

VEGF, FGF and ANGPTs transduce signals through receptor tyrosine kinases (RTKs). Spatio-temporal interactions of RTK, Notch, TGF-β, Hedgehog and WNT signaling cascades regulate a variety of processes during embryogenesis, adult-tissue homeostasis and pathogenesis, such as carcinogenesis (11). The sequential delivery of ANGPT2 and ANGPT1 using AAV effectively promotes post-ischemic angiogenesis, as ANGPT2 and ANGPT1 are involved in vascular destabilization and subsequent maturation during angiogenesis, respectively (59). A more profound understanding of the spatio-temporal interactions of regulatory signaling cascades and advances in personal genotyping and circulating miRNA profiling are mandatory to optimize the permutation and dosage of drugs for targeted therapeutics.
